# Post-meal β-cell function predicts the efficacy of glycemic control in patients with type 2 diabetes inadequately controlled by metformin monotherapy after addition of glibenclamide or acarbose

**DOI:** 10.1186/1758-5996-6-68

**Published:** 2014-05-31

**Authors:** Po-Hsun Chen, Yi-Ting Tsai, Jun-Sing Wang, Shi-Dou Lin, Wen-Jane Lee, Shih-Li Su, I-Te Lee, Shih-Te Tu, Yao-Hsien Tseng, Wayne H-H Sheu, Shih-Yi Lin

**Affiliations:** 1 Division of Endocrinology and Metabolism, Department of Internal Medicine, Taichung Veterans General Hospital, No. 1650, Sect. 4, Taiwan Boulevard, Taichung 40705, Taiwan; 2 Department of Internal Medicine, Taichung Veterans General Hospital, Chiayi branch, Chiayi, Taiwan; 3 Division of Endocrinology and Metabolism, Department of Internal Medicine, Changhua Christian Hospital, Changhua, Taiwan; 4 Department of Medical Research, Taichung Veterans General Hospital, Taichung, Taiwan; 5 School of Medicine, College of Medicine, National Yang-Ming University, Taipei, Taiwan; 6 Department of Medicine, National Defense Medical Center, Taipei, Taiwan; 7 Institute of Medical Technology, College of Life Science, National Chung-Hsing University, Taichung, Taiwan

**Keywords:** Beta-cell function, Disposition index, Glycated hemoglobin, Glycemic control, Metformin

## Abstract

**Background:**

This study aimed to explore parameters which will predict good control of HbA_1c_ after adding a second anti-diabetic drug in patients with type 2 diabetes mellitus (T2DM) inadequately controlled with metformin monotherapy.

**Methods:**

Fifty-one patients (M/F: 25/26, mean age: 53.7 ± 8.2 years, mean glycated hemoglobin [HbA_1c_] 8.4 ± 1.2%) with T2DM inadequately controlled with metformin were randomized to add-on glibenclamide or acarbose for 16 weeks. Before and after combination therapy, the subjects underwent a 2-hour liquid mixed meal tolerance test to determine insulin secretion (HOMA-β, insulinogenic index, and disposition index [DI]) and insulin sensitivity (HOMA-IR and Matsuda insulin sensitivity index).

**Results:**

At baseline, there was a significant inverse relationship between DI_120_ and HbA_1c_ (*p* = 0.001) in all subjects. The addition of glibenclamide and acarbose improved HbA_1c_ significantly from 8.6 ± 1.6% to 7.4 ± 1.2% (*p* < 0.001), and from 8.2 ± 0.8% to 7.5 ± 0.8% (*p* < 0.001), respectively. In the glibenclamide group, DI_120_ significantly increased from 51.2 ± 24.2 to 74.9 ± 41.9 (*p* < 0.05), and in the acarbose group, from 62.5 ± 31.4 to 91.7 ± 36.2 (*p* < 0.05), respectively. Multiple regression analyses showed that both baseline HbA_1c_ and DI_120_ independently predicted reduction of HbA_1c_ as well as final HbA_1c_ after combination therapy.

**Conclusions:**

In patients with T2DM inadequately controlled with metformin, add-on oral anti-diabetic agent with glibenclamide or acarbose resulted in the significant HbA_1c_ reduction and improvement of β-cell function. Subjects with greater baseline β-cell function reserve displayed better glycemic response in the combination therapy of metformin with glibenclamide or acarbose.

**Trial registration:**

This study was registered in the ClinicalTrials.gov with registration number of NCT00417729.

## Background

Impaired insulin secretion and insulin sensitivity are the main pathogenic defects in type 2 diabetes mellitus (T2DM), and can lead to either fasting or postprandial hyperglycemia [1]. The United Kingdom Prospective Diabetes Study (UKPDS) reports that at the diagnosis of T2DM, the pancreatic β-cell function is already half reduced, and then declines continuously despite the allocated therapy [2]. However, insulin insensitivity generally remains stable for years following the diagnosis [3]. Data from our group demonstrated that the contribution of PPG to glycemic control is equal to or greater than that of FPG across different ranges of HbA_1c_[4], and that this is partly accounted by the impaired early secretory defect of β-cell function in Asians, resulting in a greater contribution of PPG to overall glycemic control [5,6].

In patients with T2DM, metformin therapy is generally recommended as the first line medication for glycemic control [7]. If the patients are unable to achieve or maintain their glycemic goal, other anti-diabetic agents are usually required, but which class of drug is more suitable remains a matter of debate. It has been reported that addition of sulfonylurea or acarbose can improve glycemic control in diabetic patients who fail to reach their HbA_1c_ target with metformin alone, but limited data are available guiding the add-on class of oral anti-diabetic drug (OAD) in patients with T2DM inadequately controlled with metformin [7]. At present, few data are available to study the factors that influence the glycemic response after addition of glibenclamide and acarbose in patients with type 2 diabetes inadequately controlled by metformin monotherapy. Because β-cell dysfunction plays an important role in the progression of glycemic control in T2DM, it was hypothesized that underlying β-cell dysfunction may affect the glycemic control efficacy of the secondary added-on medication. Therefore, this study aimed to compare the clinical efficacy of addition of glibenclamide and acarbose and evaluate whether β-cell function could predict glycemic control (indicated as HbA_1c_) in patients with T2DM poorly controlled with metformin.

## Methods

### Study design

This was a 24-week, randomized, open-label, parallel study conducted at Taichung Veterans General Hospital and Changhua Christian Hospital, Taiwan. Some of the results of this study were published before [8]. In brief, outpatients with T2DM, who were 30 to 70 year-old and treated by mono- or dual- OAD therapy for above 3 months with a HbA_1c_ value of 7.0 to 11.0%, were eligible. Total 51 subjects (mean age, 54 years; females, 51%; mean body mass index (BMI), 25.6 kg/m^2^; mean HbA_1c_, 8.4% were randomized when they were inadequately controlled by metformin monotherapy (500 mg 3 times daily) for 8 weeks. Anthropometric data, FPG, HbA_1c_, and lipid profiles were measured at baseline (randomization visit) and at the end of the study after a 16-week treatment with dual oral hypoglycemic agents. Patients were excluded if they were treated with insulin or drugs that promote weight loss, had impaired renal (serum creatinine concentration >1.5 mg/dL) or liver (aspartate aminotransferase or alanine aminotransferase 2.5 times greater than the normal range) function, had a history of hemoglobinopathy or chronic anemia, or were women of child-bearing potential without adequate contraception. During the 16-week period of dual therapy, dosages were 50 mg TID for acarbose and 2.5 mg TID for glibenclamide for the first 4 weeks. For the following 12 weeks, dosages were doubled in each group, if the subjects could tolerate [8]. The present report further analyzed the relationship between HbA_1c_ and insulin secretion/sensitivity indices, and examined whether beta-cell function and insulin sensitivity were correlated with glycemic control after add-on glibenclamide or acarbose. Prior to randomization, a liquid mixed meal tolerance test (LMTT) was conducted after a 10-hour overnight fast. The liquid mixed meal contained 355.5 ml and 399 kcal (caloric contribution: 64% carbohydrate, 14% fat, and 22% protein). Blood samples were collected for measurement of serum glucose and insulin concentration at pre-meal (0 min) and at the 10^th^ min, 20^th^ min, 30^th^ min, 60^th^ min, 90^th^ min, 120^th^ min, and 180^th^ min via an indwelling venous catheter. AUC_glu_ was determined as the sum of the basal area and incremental area from 0 min to 120 min. Insulin sensitivity was estimated by homeostasis model assessment of insulin resistance (HOMA-IR) [9] and the Matsuda insulin sensitivity index (MISI) [10]. Insulin secretion was estimated by homeostasis model assessment of β-cell function (HOMA-β) [9] and the insulinogenic index calculated as the ratio of incremental insulin to glucose during the first 30 min of the LMTT (Δinsulin to Δglucose = I_30_ - I_0_/G_30_ – G_0_) [11]. In addition, because the response of insulin secretion from β-cells to hyperglycemia is modulated by the severity of insulin resistance, we also used the disposition index (DI), which is calculated as the product of insulin sensitivity and insulin secretion [12-14]: early-phase disposition index, DI_30_ = [AUC_ins 30_/AUC_glu 30_] × MISI and total disposition index, DI_120_ = [AUC_ins 120_/AUC_glu 120_] × MISI. After a 16-week therapy of dual oral hypoglycemic agents, a second LMTT was performed with all patients. The study was approved by the Institutional Review Board of Taichung Veterans General Hospital and Changhua Christian Hospital, Taiwan, and all subjects provided informed consent.

### Laboratory measurements

Plasma glucose was measured by the glucose oxidase–peroxidase method (Advia 1800; Siemens Healthcare Diagnostics Inc., Deerfield, Illinois). The inter- and intra-assay%CV for glucose were both <1.5%. Serum insulin was determined using electrochemiluminescence immunoassay (Elecsys 2010; Roche Diagnostics, Indianapolis, Indiana). The inter- and intra-assay%CV for insulin were 1.8% and 2.5%, respectively. HbA_1c_ was measured by cation-exchange HPLC (HLC-723 G7; Tosoh Bioscience Ltd., Worcestershire, United Kingdom). The inter- and intra-assay%CV for HbA_1c_ were both <4.0%.

### Statistical analyses

Data are presented as the mean ± standard deviation for continuous variables and percentage for categorical variables. The Chi-square test and Mann–Whitney *U* test were used for between-group comparison. Linear regression analyses were used to determine the relationship between any one index of insulin sensitivity or secretion and glucose control parameters, such as baseline HbA_1c_, FPG, or (AUC_glu_) in 120 min after adjustment of age, gender, baseline BMI, and disease duration. The Wilcoxon signed rank test was used to analyze the differences in BMI, FPG, HbA_1c_, HOMA-IR, HOMA-β, insulinogenic index, MISI, and DI_120_ from baseline to the end of the study. In addition, simple correlation and multiple regression analysis were conducted to evaluate the independent relationship between either HbA_1c_ level or the magnitude of HbA_1c_ reduction after combination therapy and background factors as well as baseline insulin secretion/sensitivity indices. A *p-value* of less than 0.05 was considered statistically significant. Statistical analyses were performed using SPSS version 15.0 (SPSS Inc., Chicago, Illinois).

## Results

**Figure 1:**
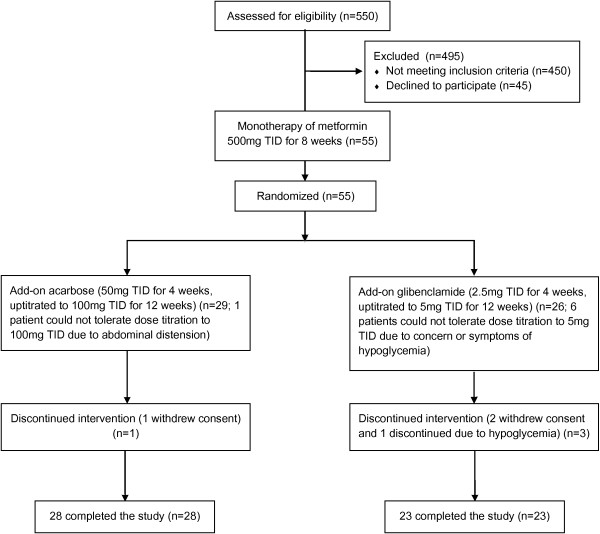
CONSORT flow diagram.

The CONSORT flow diagram of this study was shown in the Figure 1. All of the 51 subjects enrolled in the present study were treated with metformin (500 mg 3 times daily) for the first 8 weeks as a washout period. After this period, 28 subjects were treated with metformin and acarbose while another 23 were treated with metformin and glibenclamide for 16 weeks. There was no significant difference in the clinical characteristics of each group before randomization (Table 1). Multiple linear regression analyses were performed to test the association between glucose control parameters and insulin secretion/sensitivity indices after metformin monotherapy and before randomization. It was shown DI_120_ was the only parameter inversely associated with HbA_1c_ after adjustment of age, gender, disease duration, and baseline BMI. Both DI_120_ and HOMA-β significantly correlated with other glucose control parameters, FPG or AUC_glu_. As for indices of insulin sensitivity or resistance, only HOMA-IR was significantly associated with FPG (Table 2).

**Table 1:** Baseline characteristics of participants by treatment at randomization

	**All (n = 51)**	**Glibenclamide (n = 23)**	**Acarbose (n = 28)**	***P *** **value**
Gender (female, %)	51.0%	56.5%	46.4%	0.477
Age (years)	53.7 ± 8.2	54.7 ± 8.3	52.8 ± 8.2	0.378
Disease duration (years)	6.9 ± 4.6	6.0 ± 4.7	7.6 ± 4.5	0.106
BMI (kg/m^2^)	25.6 ± 3.3	25.3 ± 3.8	25.9 ± 3.0	0.334
HbA_1c_ (%)	8.4 ± 1.2	8.6 ± 1.6	8.2 ± 0.8	0.691
Fasting plasma glucose (mmol/l)	8.5 ± 2.3	9.0 ± 3.0	8.2 ± 1.3	0.538
DI_30_	41.1 ± 25.0	36.8 ± 19.1	44.7 ± 29.0	0.247
DI_120_	57.3 ± 28.6	51.2 ± 24.2	62.5 ± 31.4	0.289
HOMA-IR	3.7 ± 2.9	4.7 ± 3.9	2.9 ± 1.5	0.316
MISI	3.4 ± 1.8	3.2 ± 1.7	3.6 ± 1.8	0.321
HOMA-β (%)	44.9 ± 40.4	52.3 ± 49.7	38.9 ± 30.4	0.248
Insulinogenic index_30_ (pmol/mmol)	42.1 ± 63.4	35.0 ± 37.2	47.9 ± 79.0	0.416

Chi-square test. Mann–Whitney *U* test. Data are presented as mean ± standard deviation or percentage of participants.

DI, disposition index; HOMA-β, homeostasis model assessment β-cell function index; HOMA-IR, homeostasis model assessment insulin resistance index; MISI, Matsuda insulin sensitivity index.

**Table 2:** Multiple linear regression analysis between insulin sensitivity and secretion indices and glucose control parameters before randomization

	**HbA**_**1c**_			**Fasting plasma glucose**			**AUC**_**glu**_		
	**β**	**95% CI**	***P***	**β**	**95% CI**	***P***	**β**	**95% CI**	***P***
DI_120_	−0.784	(−0.053,-0.014)	0.001*	−0.426	(−1.053,-0.148)	0.011*	−0.525	(−14.088,-2.496)	0.006*
HOMA-β (%)	0.005	(−0.010,0.011)	0.979	−0.507	(−0.752,-0.262)	<0.001*	−0.348	(−7.040,-0.759)	0.016*
Insulinogenic index_30_ (pmol/mmol)	0.341	(−0.001,0.014)	0.075	0.211	(−0.038,0.303)	0.124	0.016	(−2.074,2.296)	0.919
HOMA-IR	−0.068	(−0.223,0.165)	0.764	0.484	(2.168,11.302)	0.005*	0.128	(−38.411,78.513)	0.492
MISI	0.270	(−0.111,0.488)	0.210	0.037	(−6.204,7.882)	0.811	−0.021	(−95.634,84.672)	0.903

Adjusted for age, gender, body mass index, and disease duration. *P < 0.05.

AUC_glu_, area under curve of glucose in 120 min; DI, disposition index; HOMA-β, homeostasis model assessment β-cell function index;

HOMA-IR, homeostasis model assessment insulin resistance index; MISI, Matsuda insulin sensitivity index.

After 16 weeks of dual-OAD therapy, there was a significant decrease in FPG and HbA_1c_ values in both groups (Table 3), and eighteen of the 51 subjects (35.3%) achieved good glycemic control of HbA_1c_ < 7.0% (9 subjects, 32.1% in acarbose group and 9 subjects, 39.1% in glibenclamide group, respectively, *p* = 0.603). Although there was no difference in HbA_1c_ between the 2 groups after add-on therapy, the mean HbA_1c_ reduction in the glibenclamide arm (1.2%) was greater than in acarbose arm (0.7%), that was compatible with the general concept that sulfonylurea has a more potent effect upon the magnitude of HbA_1c_ reduction than acarbose [7]. In addition, the insulin secretion marker, DI_120_, improved in both groups, but there was no significant difference in these insulin secretion/sensitivity surrogates, and their change before and after combination therapy between the 2 treatment groups. Multiple linear regression analyses were performed to test the relationship between baseline DI_120_ and HbA_1c_ in all subjects after combination therapy of metformin with glibenclamide or acarbose (Table 4). By using the 3 analysis models to adjust OAD classes and other possible bias factors, including age, gender, disease duration, baseline BMI, and other insulin secretion/sensitivity indices, both baseline HbA_1c_ and DI_120_ were significantly associated with HbA_1c_ after add-on therapy. Likewise, a significant association was also found between baseline DI_120_ and the magnitude of HbA_1c_ reduction after add-on therapy (Table 5). In each subgroup, simple correlation analysis showed that there was a negative correlation between baseline DI_120_ and HbA_1c_ after dual therapy in acarbose group (*r = −*0.439*, p* = 0.022), and in glibenclamide group (*r* = −0.584, *p* = 0.003), respectively.

**Table 3:** Comparison of glucose control parameters, insulin secretion and sensitivity surrogates before and after treatment in both groups

	**Acarbose (n = 28)**			**Glibenclamide (n = 23)**		
	**Before**	**After**	***P*-values vs. baseline**	**Before**	**After**	***P*-values vs. baseline**
BMI (kg/m^2^)	25.9 ± 3.0	25.5 ± 3.3	0.005*	25.3 ± 3.8	25.5 ± 4.0	0.072
Fasting plasma glucose (mmol/l)	8.2 ± 1.2	7.3 ± 1.2	0.002*	9.0 ± 3.0	7.2 ± 2.1	0.001*
HbA_1c_ (%)	8.2 ± 0.8	7.5 ± 0.8	<0.001*	8.6 ± 1.6	7.4 ± 1.2	<0.001*
HOMA-IR	3.0 ± 1.4	3.1 ± 2.9	0.682	4.8 ± 3.9	3.5 ± 2.7	0.101
HOMA-β (%)	40.3 ± 30.0	49.4 ± 40.2	0.021*	53.7 ± 50.5	47.0 ± 83.4	0.153
Insulinogenic index_30_ (pmol/mmol)	47.9 ± 79.0	50.6 ± 42.0	0.080	35.0 ± 37.2	36.1 ± 24.5	0.191
MISI	3.6 ± 1.8	4.6 ± 2.8	0.124	3.2 ± 1.7	4.1 ± 2.9	0.176
AUC_ins__120_/AUC_glu 120_^§^(pmol/mmol)	2.9 ± 2.3	3.8 ± 3.1	0.003*	2.7 ± 1.4	3.3 ± 1.7	0.121
DI_120_	62.5 ± 31.4	91.7 ± 36.2	0.002*	51.2 ± 24.2	74.9 ± 41.9	0.003*

Wilcoxon signed rank test; Data are presented as mean ± standard deviation. *P <0.05: § total area under curve of insulin within 120 minutes divided by total area under curve of glucose within 120 minutes.

DI, disposition index; HOMA-β, homeostasis model assessment β-cell function index; HOMA-IR, homeostasis model assesment insulin resistance index; MISI, Matsuda insulin sensitivity index.

**Table 4:** **Multiple linear regression models of HbA**_**1c **_**after combination therapy**

	**Model 1**		**Model 2**		**Model 3**	
	**β**	***P***	**β**	***P***	**β**	***P***
Drug group (0 = glibenclamide; 1 = acarbose)	0.089	0.410	0.152	0.127	0.145	0.126
Gender (0 = female; 1 = male)	−0.163	0.110	-	-	-	-
Age (years)	−0.106	0.307	-	-	-	-
Disease duration (years)	0.105	0.313	-	-	-	-
BMI (kg/m^2^)	0.172	0.179	-	-	-	-
HbA_1c_ (%)	0.677	<0.001*	0.687	<0.001*	0.703	<0.001*
MISI	0.162	0.391	0.217	0.234	-	-
HOMA-IR	−0.305	0.080	−0.210	0.148	−0.244	0.085
HOMA-β (%)	−0.030	0.864	0.029	0.835	-	-
Insulinogenic index_30_ (pmol/mmol)	−0.072	0.734	-	-	-	-
AUC_ins__120_/AUC_glu 120_^§^(pmol/mmol)	0.301	0.258	0.302	0.150	0.158	0.249
DI_120_	−0.801	0.045*	−0.743	0.031*	−0.552	0.030*
DI_30_	0.456	0.226	0.329	0.176	0.284	0.142

Multiple linear regression models with HbA_1c_ after combination therapy as the dependent variable. All independent variables were collected before randomization of the second-line OADs.

*P < 0.005; § total area under curve of insulin within 120 minutes divided by total area under curve of glucose within 120 minutes.

DI, disposition index; HOMA-β, homeostasis model assessment β-cell function; HOMA-IR, homeostasis model assessment insulin resistance index;

Incremental AUC, incremental area under curve during liquid mixed meal tolerance test; MISI, Matsuda insulin sensitivity index.

**Table 5:** **Multiple linear regression models of magnitude of HbA**_**1c **_**reduction after combination therapy**

	**Model 1**		**Model 2**		**Model 3**	
	**β**	***P***	**β**	***P***	**β**	***P***
Drug group (0 = glibenclamide; 1 = acarbose)	−0.113	0.410	−0.192	0.127	−0.183	0.126
Gender (0 = female; 1 = male)	0.206	0.110	-	-	-	-
Age (years)	0.134	0.307	-	-	-	-
Disease duration (years)	−0.132	0.313	-	-	-	-
BMI (kg/m^2^)	−0.217	0.179	-	-	-	-
HbA_1c_ (%)	0.730	<0.001*	0.718	<0.001*	0.698	<0.001*
MISI	−0.204	0.391	−0.273	0.234	-	-
HOMA-IR	0.384	0.080	0.265	0.148	0.308	0.085
HOMA-β (%)	0.037	0.864	−0.036	0.835	-	-
Insulinogenic index_30_ (pmol/mmol)	0.091	0.734	-	-	-	-
AUC_ins__120_/AUC_glu 120_^§^(pmol/mmol)	−0.379	0.258	−0.380	0.150	−0.199	0.249
DI_120_	1.009	0.045*	0.936	0.031*	0.696	0.030*
DI_30_	−0.574	0.226	−0.414	0.176	−0.358	0.142

Multiple linear regression models with HbA_1c_ reduction after combination therapy as the dependent variable. All independent variables were collected before randomization of the second-line OADs.

*P < 0.005; § total area under curve of insulin within 120 minutes divided by total area under curve of glucose within 120 minutes.

DI, disposition index; HOMA-β, homeostasis model assessment β-cell function; HOMA-IR, homeostasis model assessment insulin resistance index;

Incremental AUC, incremental area under curve during liquid mixed meal tolerance test; MISI, Matsuda insulin sensitivity index.

## Discussion

The main finding from the present study was that in those patients with T2DM inadequately controlled by metformin, residual β-cell function, expressed by DI_120,_ independently predicted glycemic response after adding a 2^nd^ OAD, either glibenclamide or acarbose.

It is well recognized that pancreatic β-cell dysfunction is a key pathogenetic factor involved in T2DM [1]. In 1981, Bergman et al. [15] postulated the product of insulin sensitivity and insulin secretion was a constant, namely, the disposition index (DI). This index represents the responsiveness of β-cells in compensating for insulin sensitivity [16]. In general, DI_120_ represents the overall insulin response to insulin sensitivity during oral glucose tolerance test [12]. In addition, DI_120_ derived from LMTT also has a predictive power analogous to that calculated from intravenous glucose tolerance test [13,14]. In clinical studies, DI has been shown to decrease with progression from normal glucose tolerance to diabetes mellitus, and can be used to predict the development of diabetes over a long period in a population without diabetes [17]. Of note, our study also demonstrated that before randomization, there was a significant negative association between DI_120_ and HbA_1c,_ but not HOMA-β, insulinogenic index, HOMA-IR, or MISI. These observations were supported by our previous report that PPG was an important contributor to glycemic control [4] and indicated that β-cell dysfunction relative to insulin sensitivity was a major determinant of HbA_1c_ in Asians.

There was a significant decrease in FPG and HbA_1c_ values after 16 weeks of dual-OAD therapy in both groups. It was shown that baseline HbA_1c_ was significantly associated with the magnitude of HbA_1c_ reduction after add-on therapy. Several factors, including higher baseline HbA_1c_, longer disease duration, younger age, and higher BMI have been reported to be associated with poorer glycemic control in patients with T2DM [18-20]. However, in the present study, only baseline HbA_1c_, but not age, disease duration, BMI, or gender, independently predicted good glycemic control after adding glibenclamide or acarbose to metformin therapy. It was speculated that a small sample size, short follow-up duration, and limited OAD classes might be causes of inconsistent results.

In addition to decreased HbA_1c_ after add-on second OAD, the insulin secretion marker, DI_120_, also improved in both groups. It is proposed that ameliorating hyperglycemia in subjects with type 2 diabetes might also have a helpful effect on β-cell failure by attenuating so-called glucose toxicity effect [21]. Particularly, our study also found that baseline DI_120_ was an independent predictor of glycemic control after adding glibenclamide or acarbose in the subjects inadequately controlled by metformin monotherapy. It has been reported that β-cell dysfunction can relate to HbA_1c_ in newly diagnosed T2DM [22,23], or in already OAD-treated adults with T2DM [24-26]. However, some of these studies were limited in that they disclosed only the cross-sectional relationship between β-cell function and glycemic parameters. Our study findings would extend the role of β-cell function in predicting the therapeutic response of HbA_1c_ levels. This result may be particularly important in Asian people with diabetes because it is proposed that β-cell dysfunction plays a major role in the pathogenesis of T2DM in this group of patients [5], and thus can determine HbA_1c_ response after use of oral hypoglycemic agents.

Several studies have reported that the addition of sulfonylurea or acarbose improved glycemic control in patients with T2DM unable to achieve or maintain glycemic control with metformin monotherapy alone [7,27]. It is generally accepted that sulfonylureas exert their hypoglycemic effect in part through direct action on pancreatic β-cells, which augments insulin secretion, although improvements in insulin sensitivity have also been reported in some, but not all studies [28-30]. Based on the anti-hyperglycemic mechanisms of sulfonylurea, it seemed reasonable that patients with higher baseline DI_120_ would have a better treatment response after adding glibencalmide, as seen in our patients.

Alpha-glucosidase inhibitors, such as acarbose, act via inhibiting disaccharide hydrolyzing enzymes in the small intestine, thereby decreasing glucose absorption and improving control over postprandial hyperglycemia. Acarbose has also been found to improve both insulin resistance and secretion indirectly in obese patients with T2DM [31,32]. It is postulated that insulin secretion and sensitivity might have improved at least in part through a decrease in glucose toxicity, because α-glucosidase inhibitors do not have a direct effect on insulin secretion or sensitivity. It was speculated that the improvement in HbA_1c_ in the patients in the acarbose group was a result of cooperative improvement of insulin sensitivity and secretion after an amelioration of glucose toxicity, and thus residual β-cell function still played a role in the regulation of glycemic response after acarbose [33].

The strength of our study is the collection of insulin secretion or insulin sensitivity parameters during LMTT, which is not easily done in clinical studies. However, this LMTT was limited by that some subjects still used the investigational medication (e.g. sulfonylurea) in the evening before the 2^nd^ LMTT, and thus the DI_120_ derived from the 2^nd^ LMTT may reflect drug-stimulated rather than endogenous residual β-cell function. Second, our study enrolled small sample size of patients a single ethnic population, which might influence statistical power to analyze whether DI_120_ was a significant predictor of HbA_1c_ after combination therapy of metformin with glibenclamide or acarbose. Third, this study had short washout period, that might make the baseline HbA_1c_ at inclusion underestimated in patients taking sulfonylurea in comparison to those selected after metformin monotherapy failure. Finally, we did not evaluate the other choices of add-on medication. Further prospective studies with more patients and longer follow-up are needed to determine the association between DI_120_ and good glycemic control, especially in connection with different OADs.

## Conclusions

Addition of glibenclamide or acarbose resulted in the significant HbA_1c_ reduction and improvement of DI_120_ in patients poorly controlled with metformin, and post-meal DI_120_ predicted the change in HbA_1c_ in each group after addition of glibenclamide or acarbose. It is suggested that residual β-cell function reserve may help to predict glycemic control response of combination of these agents.

## Abbreviations

AUC_glu_: Area under curve of glucose; AUC_ins_: Area under curve of insulin; BMI: Body mass index; DI: Disposition index; FPG: Fasting plasma glucose; HbA1c: Glycated hemoglobin; HOMA-IR: Homeostasis model assessment insulin resistance index; HOMA-β: Homeostasis model assessmentβ-cell function index; LMTT: Liquid mixed meal tolerance test; MISI: Matsuda insulin sensitivity index; OAD: Oral anti-diabetic drug; PPG: Postprandial glucose; T2DM: Type 2 diabetes mellitus; UKPDS: United Kingdom prospective diabetes study.

## Competing interests

I-T Lee received grants from MSD and Bayer Schering Pharma. S-T Tu has been a consultant for MSD, Bayer Schering Pharma, Eli Lilly, Astra-Zeneca, and BMS, and received honoraria from MSD, Bayer Schering Pharma, Eli Lilly, BMS, and Novo-Nordisc; he has also received grants from Bayer Schering Pharma. Wayne H-H Sheu has been a consultant for MSD, Roche, Bayer Schering Pharma, Eli Lilly, Astra-Zeneca, and BMS, and received honoraria from MSD, Roche, Bayer Schering Pharma, Eli Lilly, Astra-Zeneca, BMS, and Novo-Nordisc; he has also received grants from MSD and Bayer Schering Pharma. The other authors whose names are listed above certify that they have NO affiliations with or involvement in any organization or entity with any financial interest (such as honoraria; educational grants; participation in speakers’ bureaus; membership, employment, consultancies, stock ownership, or other equity interest, and expert testimony or patent-licensing arrangements), or non-financial interest (such as personal or professional relationships, affiliations, knowledge or beliefs) in the subject matter or materials discussed in this manuscript.

## Authors’ contributions

P-HC interpreted the data and wrote the manuscript; Y-TT interpreted the data and wrote the manuscript; J-SW conducted the study and performed the data collection; S-DL conducted the study; W-JL conducted the study and performed the data collection; S-LS conducted the study; I-TL conducted the study and performed data collection; S-TT conducted the study; Y-HT conducted the study; WH-HS conducted the study and interpreted the data; S-YL conducted the study, interpreted the data, and wrote the manuscript. All authors read and approved the final manuscript.

## References

[B1] DeFronzo RA Pathogenesis of type 2 diabetes mellitus Med Clin North A 2004 88 787 835 ix 10.1016/j.mcna.2004.04.013 15308380

[B2] U.K. Prospective diabetes study group U.K. Prospective diabetes study 16. Overview of 6 years’ therapy of type II diabetes: a progressive disease Diabetes 1995 44 1249 1258 7589820

[B3] Levy J Atkinson AB Bell PM McCance DR Hadden DR Beta-cell deterioration determines the onset and rate of progression of secondary dietary failure in type 2 diabetes mellitus: the 10-year follow-up of the Belfast Diet Study Diabet Med 1998 15 290 296 10.1002/(SICI)1096-9136(199804)15:4<290::AID-DIA570>3.0.CO;2-M 9585393

[B4] Wang JS Tu ST Lee IT Lin SD Lin SY Su SL Lee WJ Sheu WH Contribution of postprandial glucose to excess hyperglycaemia in Asian type 2 diabetic patients using continuous glucose monitoring Diabetes Metab Res Rev 2011 27 79 84 10.1002/dmrr.1149 21218511

[B5] Chan JC Malik V Jia W Kadowaki T Yajnik CS Yoon KH Hu FB Diabetes in Asia: epidemiology, risk factors, and pathophysiology JAMA 2009 301 2129 2140 10.1001/jama.2009.726 19470990

[B6] Fukushima M Suzuki H Seino Y Insulin secretion capacity in the development from normal glucose tolerance to type 2 diabetes Diabetes Res Clin Pract 2004 66 Suppl 1 S37 S43 15563978 10.1016/j.diabres.2003.11.024

[B7] Inzucchi SE Bergenstal RM Buse JB Diamant M Ferrannini E Nauck M Peters AL Tsapas A Wender R Matthews DR American Diabetes A, European Association for the Study of D Management of hyperglycemia in type 2 diabetes: a patient-centered approach: position statement of the American Diabetes Association (ADA) and the European Association for the Study of Diabetes (EASD) Diabetes Care 2012 35 1364 1379 10.2337/dc12-0413 22517736 PMC3357214

[B8] Wang JS Lin SD Lee WJ Su SL Lee IT Tu ST Tseng YH Lin SY Sheu WH Effects of acarbose versus glibenclamide on glycemic excursion and oxidative stress in type 2 diabetic patients inadequately controlled by metformin: a 24-week, randomized, open-label, parallel-group comparison Clin Ther 2011 33 1932 1942 10.1016/j.clinthera.2011.10.014 22078152

[B9] Matthews DR Hosker JP Rudenski AS Naylor BA Treacher DF Turner RC Homeostasis model assessment: insulin resistance and beta-cell function from fasting plasma glucose and insulin concentrations in man Diabetologia 1985 28 412 419 10.1007/BF00280883 3899825

[B10] Matsuda M DeFronzo RA Insulin sensitivity indices obtained from oral glucose tolerance testing: comparison with the euglycemic insulin clamp Diabetes Care 1999 22 1462 1470 10.2337/diacare.22.9.1462 10480510

[B11] Phillips DI Clark PM Hales CN Osmond C Understanding oral glucose tolerance: comparison of glucose or insulin measurements during the oral glucose tolerance test with specific measurements of insulin resistance and insulin secretion Diabet Med 1994 11 286 292 10.1111/j.1464-5491.1994.tb00273.x 8033528

[B12] Cobelli C Toffolo GM Dalla Man C Campioni M Denti P Caumo A Butler P Rizza R Assessment of beta-cell function in humans, simultaneously with insulin sensitivity and hepatic extraction, from intravenous and oral glucose tests Am J Physiol Endocrinol Metab 2007 293 E1 E15 10.1152/ajpendo.00421.2006 17341552

[B13] Maki KC Rains TM Dicklin MR Bell M Repeatability of indices of insulin sensitivity and secretion from standard liquid meal tests in subjects with type 2 diabetes mellitus or normal or impaired fasting glucose Diabetes Technol Ther 2010 12 895 900 10.1089/dia.2010.0083 20879960

[B14] Maki KC Kelley KM Lawless AL Hubacher RL Schild AL Dicklin MR Rains TM Validation of insulin sensitivity and secretion indices derived from the liquid meal tolerance test Diabetes Technol Ther 2011 13 661 666 10.1089/dia.2010.0240 21457067

[B15] Bergman RN Phillips LS Cobelli C Physiologic evaluation of factors controlling glucose tolerance in man: measurement of insulin sensitivity and beta-cell glucose sensitivity from the response to intravenous glucose J Clin Invest 1981 68 1456 1467 10.1172/JCI110398 7033284 PMC370948

[B16] Bergman RN Ader M Huecking K Van Citters G Accurate assessment of beta-cell function: the hyperbolic correction Diabetes 2002 51 Suppl 1 S212 S220 11815482 10.2337/diabetes.51.2007.s212

[B17] Utzschneider KM Prigeon RL Faulenbach MV Tong J Carr DB Boyko EJ Leonetti DL McNeely MJ Fujimoto WY Kahn SE Oral disposition index predicts the development of future diabetes above and beyond fasting and 2-h glucose levels Diabetes Care 2009 32 335 341 18957530 10.2337/dc08-1478 PMC2628704

[B18] Giugliano D Maiorino M Bellastella G Chiodini P Esposito K Relationship of baseline HbA1c, HbA1c change and HbA1c target of < 7% with insulin analogues in type 2 diabetes: a meta-analysis of randomised controlled trials Int J Clin Pract 2011 65 602 612 10.1111/j.1742-1241.2010.02619.x 21489084

[B19] Khattab M Khader YS Al-Khawaldeh A Ajlouni K Factors associated with poor glycemic control among patients with type 2 diabetes J Diabetes Complications 2010 24 84 89 10.1016/j.jdiacomp.2008.12.008 19282203

[B20] Benoit SR Fleming R Philis-Tsimikas A Ji M Predictors of glycemic control among patients with Type 2 diabetes: a longitudinal study BMC Public Health 2005 5 36 10.1186/1471-2458-5-36 15833140 PMC1090595

[B21] Weir GC Bonner-Weir S Five stages of evolving beta-cell dysfunction during progression to diabetes Diabetes 2004 53 Suppl 3 S16 S21 15561905 10.2337/diabetes.53.suppl_3.s16

[B22] Cernea S Huţanu A Coroş L Dobreanu M Assessment of beta cell function in subjects with newly diagnosed type 2 diabetes Revista Română de Medicină de Laborator 2013 21 145 160

[B23] Albarrak AI Luzio SD Chassin LJ Playle RA Owens DR Hovorka R Associations of glucose control with insulin sensitivity and pancreatic beta-cell responsiveness in newly presenting type 2 diabetes J Clin Endocrinol Metab 2002 87 198 203 11788647 10.1210/jcem.87.1.8152

[B24] Dansuntornwong B Chanprasertyothin S Jongjaroenprasert W Ngarmukos C Bunnag P Puavilai G Ongphiphadhanakul B The relation between parameters from homeostasis model assessment and glycemic control in type 2 diabetes J Med Assoc Thai 2007 90 2284 2290 18181308

[B25] Kohnert KD Augstein P Zander E Heinke P Peterson K Freyse EJ Hovorka R Salzsieder E Glycemic variability correlates strongly with postprandial beta-cell dysfunction in a segment of type 2 diabetic patients using oral hypoglycemic agents Diabetes Care 2009 32 1058 1062 10.2337/dc08-1956 19244086 PMC2681045

[B26] Shim WS Kim SK Kim HJ Kang ES Ahn CW Lim SK Lee HC Cha BS Decrement of postprandial insulin secretion determines the progressive nature of type-2 diabetes Eur J Endocrinol 2006 155 615 622 10.1530/eje.1.02249 16990662

[B27] Phillips P Karrasch J Scott R Wilson D Moses R Acarbose improves glycemic control in overweight type 2 diabetic patients insufficiently treated with metformin Diabetes Care 2003 26 269 273 10.2337/diacare.26.2.269 12547847

[B28] Simonson DC Ferrannini E Bevilacqua S Smith D Barrett E Carlson R DeFronzo RA Mechanism of improvement in glucose metabolism after chronic glyburide therapy Diabetes 1984 33 838 845 10.2337/diab.33.9.838 6432610

[B29] Kolterman OG Gray RS Shapiro G Scarlett JA Griffin J Olefsky JM The acute and chronic effects of sulfonylurea therapy in type II diabetic subjects Diabetes 1984 33 346 354 10.2337/diab.33.4.346 6423429

[B30] Beck-Nielsen H Hother-Nielsen O Pedersen O Mechanism of action of sulphonylureas with special reference to the extrapancreatic effect: an overview Diabet Med 1988 5 613 620 10.1111/j.1464-5491.1988.tb01068.x 2975544

[B31] Delgado H Lehmann T Bobbioni-Harsch E Ybarra J Golay A Acarbose improves indirectly both insulin resistance and secretion in obese type 2 diabetic patients Diabetes Metab 2002 28 195 200 12149599

[B32] Meneilly GS Ryan EA Radziuk J Lau DC Yale JF Morais J Chiasson JL Rabasa-Lhoret R Maheux P Tessier D Wolever T Josse RG Elahi D Effect of acarbose on insulin sensitivity in elderly patients with diabetes Diabetes Care 2000 23 1162 1167 10.2337/diacare.23.8.1162 10937515

[B33] Rosak C Hofmann U Paulwitz O Modification of beta-cell response to different postprandial blood glucose concentrations by prandial repaglinide and combined acarbose/repaglinide application Diabetes Nutr Metab 2004 17 137 142 15334790

